# Sunset of you

**DOI:** 10.1017/S1478951526102855

**Published:** 2026-06-10

**Authors:** Miguel Julião

**Affiliations:** Equipa Comunitária de Suporte em Cuidados Paliativos PalCo, ULS Amadora/Sintra, Amadora, Portugal

when I let you slip away from me,
closing the windows of our place,
I knew I could never touch you again.
you became my golden sunset… [only]

and now…

I see, from afar, your hair gathered up,
I smell, from afar, the unspeakable scent of your nape.
I have not lost you, yet I do not have you hand in hand,
fingers entwined in the car,
our music playing in our heads.

and now…
every day…

I lean against the closed window [I dare not open]
I think of you,
I feel you only in every exchanged glance.
a smile passing by,
far away, though so close.

and every day I wait
for a boat to come for me,
sailing me to the edge of the horizon,
where your eyes,
your hair,
and the perfume of your nape
paint the most beautiful sunset on our canvas,
beneath the blue of the sky.

and I hope, by the window, we will embrace again,
and that the window becomes a door,
and that door stands within our walls –
at last, our place,
where we lay down and entwine our fingers.

